# Development and Validation of Clinical Prediction Models for Surgical Success in Patients With Endometriosis: Protocol for a Mixed Methods Study

**DOI:** 10.2196/20986

**Published:** 2021-04-05

**Authors:** Nadine Marlin, Carol Rivas, John Allotey, Julie Dodds, Andrew Horne, Elizabeth Ball

**Affiliations:** 1 Pragmatic Clinical Trials Unit Centre for Primary Care and Public Health Queen Mary University of London London United Kingdom; 2 UCL Social Research Institute University College London London United Kingdom; 3 Women's Health Research Unit Centre for Primary Care and Public Health Queen Mary University of London London United Kingdom; 4 MRC Centre for Reproductive Health University of Edinburgh Edinburgh United Kingdom

**Keywords:** endometriosis, algorithm, laparoscopy, pain, therapeutic

## Abstract

**Background:**

Endometriosis is a chronic inflammatory condition affecting 6%-10% of women of reproductive age and is defined by the presence of endometrial-like tissue outside the uterus (lesions), commonly affecting the pelvis and ovaries. It is associated with debilitating pelvic pain, infertility, and fatigue and often has devastating effects on the quality of life (QoL). Although it is as common as back pain, it is poorly understood, and treatment and diagnosis are often delayed, leading to unnecessary suffering. Endometriosis has no cure. Surgery is one of several management options. Quantifying the probability of successful surgery is important for guiding clinical decisions and treatment strategies. Factors predicting success through pain reduction after endometriosis surgery have not yet been adequately identified.

**Objective:**

This study aims to determine which women with confirmed endometriosis benefit from surgical improvement in pain and QoL and whether these women could be identified from clinical symptoms measured before laparoscopy.

**Methods:**

First, we will carry out a systematic search and review and, if appropriate, meta-analysis of observational cohort and case-control studies reporting one or more risk factors for endometriosis and postsurgical treatment success. We will search PubMed, Embase, and Cochrane databases from inception without language restrictions and supplement the reference lists by manual searches. Second, we will develop separate clinical prediction models for women with confirmed and suspected diagnoses of endometriosis. A total of three suitable databases have been identified for development and external validation (the MEDAL [ISRCTN13028601] and LUNA [ISRCTN41196151] studies, and the BSGE database), and access has been guaranteed. The models will be developed using a linear regression approach that links candidate factors to outcomes. Third, we will hold 2 stakeholder co-design workshops involving eight clinicians and eight women with endometriosis separately and then bring all 16 participants together. Participants will discuss the implementation, delivery, usefulness, and sustainability of the prediction models. Clinicians will also focus on the ease of use and access to clinical prediction tools.

**Results:**

This project was funded in March 2018 and approved by the Institutional Research Ethics Board in December 2019. At the time of writing, this study was in the data analysis phase, and the results are expected to be available in April 2021.

**Conclusions:**

This study is the first to aim to predict who will benefit most from laparoscopic surgery through the reduction of pain or increased QoL. The models will provide clinicians with robustly developed and externally validated support tools, improving decision making in the diagnosis and treatment of women.

**International Registered Report Identifier (IRRID):**

DERR1-10.2196/20986

## Introduction

### Background on Endometriosis

Endometriosis is a chronic inflammatory condition affecting 6%-10% of women of reproductive age. It is defined by the presence of endometrial-like tissue outside the uterus (lesions), commonly affecting the pelvis and ovaries [1,2]. Although as common as back pain, it is poorly understood, and treatment and diagnosis are often delayed [2]. For example, in the United Kingdom, there is an average delay of 7-9 years in accessing treatment for endometriosis [3]. This is largely due to the lack of accurate, noninvasive diagnostic tests or biomarkers [4]. The diagnostic gold standard is pelvic laparoscopy under general anesthesia. Laparoscopy can be diagnostic, therapeutic, or both.

Diagnostic delays lead to unnecessary suffering, and endometriosis is associated with debilitating pelvic pain, infertility, and fatigue and can have devastating effects on the quality of life (QoL). Endometriosis has no cure, but there are a number of treatment options. These include drugs that suppress ovarian function (which can have adverse effects [5]) or surgery for the lesions (usually laparoscopically). Surgical removal is often considered the best option for symptomatic endometriosis [6], but it does not reduce pain in 20%-28% of patients who undergo surgery [7,8].

### Variable Response to Surgery

Quantifying the potential for improvement in a woman’s symptoms after surgery is important for guiding clinical decisions and treatment strategies. Secondary findings from observational, single-center studies indicate a graded response regarding pain reduction after endometriosis surgery, which is inversely related to disease severity [8-11]. One randomized controlled trial (RCT) found that pain symptoms improved after endometriosis surgery in significantly more patients with moderate and mild endometriosis (approximately 100% and 70%, respectively) than minimal disease (approximately 40%) [9]. In 2 other studies, women with deep endometriosis (DE) experienced more pain reduction after surgery than those with superficial endometriosis [10,11].

Although National Health Service (NHS) England recommends that women should undergo therapeutic laparoscopy for complicated DE in specialist endometriosis centers, we have no evidence-based information to recognize these women clinically. Nor are we able, at present, to recognize which women will respond to this treatment. As a result, therapeutic laparoscopy, a costly and limited resource with long waiting lists, is not necessarily carried out on those who will experience pain reduction.

Factors predicting pain reduction after endometriosis surgery have not yet been adequately identified, as they have never been studied as the primary research question. However, secondary outcomes from previous trials indicate that such factors can be identified [8,9,11]. Our aim is to address the existing gap by predicting success through pain reduction after endometriosis surgery.

## Methods

The CRESCENDO (Creating a Clinical Prediction Model to predict Surgical Success in Endometriosis) project will be undertaken using existing recommendations for prognostic research model development, validation [12-14], and reporting [15]. It will also involve a systematic review of clinical risk factors associated with endometriosis and postsurgery treatment success, which will adhere to the PRISMA (Preferred Reporting Items for Systematic Reviews and Meta-Analyses) guidelines [16]. The systematic review is registered in the International Prospective Register of Systematic Reviews (PROSPERO).

### Aim

To develop clinical prediction models for endometriosis to answer 2 fundamental questions regarding the surgical management of endometriosis:

1. Which women with confirmed endometriosis benefit from surgery and see improvement in pain and QoL? (Primary models)
2. Could these women be identified based on clinical symptoms measured before laparoscopy? (Secondary models)

### Specific Objectives

1. To perform a systematic review of preoperative and intraoperative factors associated with postsurgical treatment success in endometriosis.
2. To perform a systematic review of the clinical risk factors associated with endometriosis.
3. To develop and validate clinical prediction models to predict changes in self-reported pain and QoL after surgery in women with a confirmed (primary models) or suspected (secondary models) diagnosis of endometriosis.
4. To describe an implementation plan within the secondary care pathway for women with a confirmed or suspected diagnosis of endometriosis using co-design workshops.

### Systematic Review

We will carry out a systematic review and meta-analysis of observational cohort and case-control studies reporting 1 or more risk factors for endometriosis and predictors of postsurgical treatment success. We aim to determine the following:

- Absolute risk of having endometriosis in the presence or absence of a given risk factor.
- Relative risk of having endometriosis in the presence or absence of a given risk factor.
- The population attributable fraction for endometriosis in relation to each risk factor.
- Pre- and intraoperative factors for postsurgical treatment success for endometriosis and the associated risks.

#### Literature Search

We will search PubMed, Embase, and Cochrane databases from inception without any language restrictions and supplement these with manual searches of reference lists of included primary studies and relevant review articles.

#### Study Selection, Data Extraction, and Quality Assessment

The first reviewers will independently screen the titles and abstracts to identify the eligible studies, followed by retrieval and assessment of full texts of potentially relevant articles. Any disagreement will be resolved following discussions with a different reviewer. We will extract data in duplicate using predesigned data extraction forms. We will assess the quality of methodology of the included studies using the Newcastle Ottawa Scale [17] or the Jadad score [18], depending on the study.

### Data Sets for Development of the Clinical Prediction Models

We have identified 3 suitable data sets that will be employed to develop and validate the clinical prediction models for women with confirmed and suspected diagnoses of endometriosis. We have guaranteed access to all three databases and data sharing agreements have been finalized *a priori*.

The British Society of Gynaecological Endoscopy (BSGE) maintains a national database that contains data of over 5000 women who had a confirmed diagnosis and underwent laparoscopic surgery for advanced endometriosis (stage 4). Records have been collected by clinicians at over 50 endometriosis centers in the United Kingdom since 2007 (data collection is ongoing). Endometriosis centers are commissioned by NHS England and accredited by the BSGE for complex multidisciplinary surgery required for the treatment of DE [19,20]. To maintain accreditation, the database serves as a mandatory record of DE cases and outcomes [21]. It includes data on patient characteristics, pain, and QoL before and 6, 12, and 24 months after surgery, along with intraoperative findings. It is the most comprehensive source of data on endometriosis surgery and QoL worldwide. Some of these data have recently been published [22].

The second data set comes from a clinical study of women with suspected endometriosis, specifically chronic pelvic pain. *MRI versus laparoscopy to diagnose the main causes of chronic pelvic pain in women: a test-accuracy study and economic evaluation* (MRI to establish diagnosis against laparoscopy [MEDAL]) [ISRCTN13028601], was a comparative test-accuracy study assessing whether magnetic resonance imaging (MRI) could replace or triage the use of laparoscopy in establishing a diagnosis among women presenting in secondary care with chronic pelvic pain. Data were collected on patient characteristics, pain, and QoL before and after diagnostic laparoscopy with or without surgery, along with intraoperative findings for over 300 women who underwent laparoscopic surgery for chronic pelvic pain at 26 UK hospitals. During surgery, over a third of the women were diagnosed with endometriosis [23].

The third data set included women with suspected endometriosis collected during an RCT. This study, *Laparoscopic Uterosacral Nerve Ablation (LUNA) for alleviating chronic pelvic pain* [ISRCTN41196151]*,* randomized 487 women with chronic pelvic pain from 18 UK hospitals to assess the effectiveness of laparoscopic uterine nerve ablation. No significant improvements were reported on the visual analog pain scales. The data collected were similar to MEDAL and included patient characteristics, pain, and QoL at multiple time points [24].

### Establishment of Data Sharing

The Queen Mary University of London pragmatic clinical trials unit (PCTU) will provide data management for secure data set transfer and storage in accordance with general data protection regulation and information governance principles. Data will be stored within a PCTU safe haven. No additional data collection will be needed. The MEDAL, LUNA, and BSGE data will be given to the PCTU in a pseudonymized form. The minimum data to be collected from the 3 data sets will be agreed upon by the study team, collaborators, and study steering committee. All recorded variables will be considered for collection.

### Data Preparation

Data will be supplied in the format convenient for the original researchers. This project will take responsibility for converting, cleaning, and formatting the data as required before analysis. MEDAL and LUNA are data sets from previous funded studies and have been quality checked, analyzed, and published in peer-reviewed journals. Therefore, we will not assess the quality of the data, and we expect a limited need to clean them. Analyses of the BSGE data set have recently been published [22]. However, we will receive the raw source data, which will require cleaning, data quality checks, and assessment of the availability of relevant data for inclusion in the analysis. The collection of BSGE data is ongoing, so matching results from previous publications will be limited.

The 3 data sets (BSGE, MEDAL, and LUNA) will be employed when creating the prediction models, either as *development* or *external validation* data sets. To enable external validation of the prediction models, the predictors in the development data set will be matched with the variables in the validation data set. Where a direct match is not available in the data, we will investigate whether a new variable can be created from other information, such as calculating BMI from weight and height or categorizing continuous variables into groups.

### Outcomes

Treatment success will be defined by changes in self-reported pain scores or QoL from baseline to 6 months or 1 year after surgery. Three months was considered too short, because there could be a placebo effect from the surgery [25]. One year was chosen as optimal for ensuring that postoperative healing was complete, and periods had returned (some women are given medication preoperatively to stop periods); the 6-month follow-up was included for pragmatic reasons as it is the longest follow-up duration many studies achieve, as ascertained in initial scoping work. All three data sets have collected outcomes at the time points that enable this. Self-reported pain has been recorded in the data sets on a visual analog scale (VAS; score 0-10) and includes a range of specific pain symptoms such as dysmenorrhea (painful periods) and less specific symptoms such as chronic, noncyclical pelvic pain. QoL will be assessed using the EuroQol-5 Dimension questionnaire and a VAS on the overall health state (score 0-100).

After extensive discussions between the study team and the patient and public involvement (PPI) group as well as interested clinicians, the most clinically relevant outcomes, to be determined from the data on women with menstrual cycles but also potentially relevant for those without them, have been chosen to be as follows:

1. Pain-dysmenorrhea
2. Pain-dyspareunia
3. Pain-chronic pelvic pain
4. Pain-dyschezia
5. QoL-overall health state

The PPI group and clinicians have also indicated a strong preference for a separate prediction model for each outcome instead of using a composite of multiple pain measurements. This will allow clinicians and patients to predict treatment success for a patient’s specific pain profile. All outcomes will be predicted on a continuous scale, rather than dichotomizing the change in score using arbitrary cut-offs.

In the MEDAL database, symptom duration is defined as the average level of pain over the last month. The BSGE database also records women’s self-rating of their pain over the last cycle. As these data are used to build the models, we suggest that the pain types that are input into the model are assessed over the same timescale. Women in the MEDAL study were followed up for 6 months. Hence, the computed output of change in pain is necessary for the timepoint of 6 months.

### Candidate Factors

A list of candidate factors will be finalized before model development begins. Different factors might be considered in the analysis of treatment success in women with confirmed and suspected endometriosis.

Candidate factors will be identified through expert clinical input as well as through a systematic review. We will use the single factors that were investigated in narrative systematic reviews [19,26] and factors that were used as a part of previous models. These are likely to overlap. We will confirm that any candidate factor is available in the data for the analysis.

### Sample Size Considerations

This study uses pre-existing data sets to develop and validate multivariable prediction models, and at the outset of this project, no formal guidance on the minimum sample size was available. The available records from all 3 data sets will be used. However, for confirmation and after completing the analysis, we will compare our results with recently published recommendations for sample size calculations in prognostic studies [27].

The BSGE data set contains records of approximately 5000 women, of which approximately one-third are complete. Data availability for analysis is likely to increase with the selection of candidate factors from the list of available variables.

The MEDAL data set contains records of over 300 women, with approximately 110 confirmed diagnoses of endometriosis. We assume no impact of the additional diagnostic test (MRI) performed in one arm of the study and therefore include the full study population in our analyses. The analysis of treatment success in women with suspected endometriosis will examine a reduced set of candidate factors that are available before diagnostic laparoscopy. We will ensure that the list of factors considered is appropriate for the available sample size.

The LUNA data set will serve as an external validation set for the model developed to predict treatment success in women with suspected endometriosis. We will include both the treatment and control arms of the trial, as no evidence of an effect of the LUNA intervention was found for any of the pain outcomes included. The LUNA data set contains records of over 590 women, with approximately 140 women with confirmed endometriosis.

### Model Development

A total of 2 groups of models will be developed, reflecting the 2 different populations of women we aim to study. The group of 5 primary models will predict treatment success in women with confirmed diagnoses according to the 5 outcomes described above. The second group will consist of 5 models that predict treatment success in women with suspected endometriosis. Overall, this will result in 10 distinct models.

For the primary models (for women with a confirmed diagnosis of endometriosis), we will use the BSGE data set; a randomly selected 10% of records will be removed for performance testing of the model. For the secondary models of women with suspected diagnoses, we will use the MEDAL data set. All models will be developed using a logistic regression approach, linking candidate factors to outcomes.

A backward selection process will be used to decide which of the candidate predictor variables should be included in the final models (with a cutoff value of *P*<.15 conservatively taken to warrant inclusion and prevent omission of important predictors); candidate factors will likely vary between the models.

Continuous variables will be kept as continuous in the models (and not dichotomized) to avoid loss of power. Nonlinear effects may be considered if allowed by the sample size. No imputation of missing data will be performed, but the missing data at random assumption will be investigated.

### Model Performance and Internal Validation

After developing the models, we will assess how well they perform. Calibration will be assessed visually using scatter plots; the calibration slope will indicate whether predictions are systematically too high or low (calibration-in-the-large). The apparent performance of the final models will be evaluated in terms of discrimination using the C statistic.

As apparent performance is often optimistic (due to a model being developed and validated in the same data set), internal validation, which we refer to as model performance, will also be undertaken. For the primary models of treatment success in women with confirmed endometriosis, we will randomly divide the BSGE records into 2 data sets, development (90% of the data) and validation (10% of the data). The apparent performance of the models in the validation sample will be compared with their performance in the development data set. *Optimism* is the difference between the apparent value in the validation sample and the observed value in the development data set. This optimism estimate is then subtracted from the model’s apparent performance to obtain an optimism-adjusted estimate of each measure of performance for the model.

The sample size in the MEDAL data set is not sufficient to use the same approach for the secondary models of treatment success in women with suspected endometriosis. If deemed appropriate, we may implement a bootstrap resampling technique whereby the apparent performance of the developed model in bootstrap samples is compared with its performance in the model developed using the original data set.

### External Validation and Recalibration

In the last step, we will assess how well our models work when transferred into another setting (using another database).

The models of treatment success in women with confirmed endometriosis (ie, those developed in BSGE) will be externally validated in the MEDAL data set, and we will validate the models of treatment success in women with suspected endometriosis (ie, the ones developed in MEDAL) in the LUNA data set.

We will plot the agreement between observed and predicted change scores and assess calibration-in-the-large across deciles. In terms of discrimination, we will calculate the C statistic and its CI. We may consider updating the models, if they show poor performance in adjusting to the new situation, by carrying out recalibration or revision depending on discrimination and calibration performance. Table 1 shows how each data set will be used during these steps.

**Table 1:** CRESCENDO (Creating a Clinical Prediction Model to predict Surgical Success in Endometriosis) data sets and their planned use during analysis^a^.

Analysis step and primary models: treatment success in women with confirmed diagnosis		Secondary models: treatment success in women with suspected endometriosis
**Model development**		
	Random 90% of BSGE^b^ data set	MEDAL^c^ data set
**Model performance**		
	Remaining 10% of BSGE data set	Bootstrapped samples of MEDAL data set or another appropriate method
**External validation**		
	MEDAL data set	Laparoscopic uterosacral nerve ablation (control arm only)

^a^Databases containing pre-, intra-, and postoperative information of women with deep endometriosis (British Society of Gynaecological Endoscopy) or absent or superficial endometriosis (magnetic resonance imaging to establish diagnosis against laparoscopy or laparoscopic uterosacral nerve ablation).

^b^BSGE: British Society of Gynaecological Endoscopy.

^c^MEDAL: magnetic resonance imaging to establish diagnosis against laparoscopy.

### Presentation of the Prediction Models

The final step of model development will be the translation of the models into easy-to-calculate scores. These scores will be presented against observed change scores, and if appropriate, they will be grouped by categories of treatment success. These categories may be defined in line with usual practice (eg, improvement by at least 1 point on VAS or change by less than 1 point on VAS or deterioration by at least 1 point on VAS). Descriptive comparisons will be presented for women in different categories of treatment success.

### Stakeholder Co-design Workshops

Facilitated stakeholder co-design workshop discussions will center around a video made by the study team beforehand. The video will comprise 4 to 5 women, recruited as part of the study process, recounting their endometriosis treatment decision-making experience. Participants will be asked to identify and discuss key *touch-points*, that is, points within the endometriosis pathway where our clinical prediction model might have an emotional or clinical impact or where there may be impediments to its sustainable implementation. We will conceal patient identities in the video if they require this, for example, by masking their faces or having a member of the study team recount their experience on the video from a transcription.

A total of three workshops will be held. The first will involve 8 clinicians; in the second, we will work with 8 women with endometriosis; and in the third, we will bring all 16 participants together. Each workshop will last 2 h and all are planned to be on the same day, with the first 2 being simultaneous. We currently envisage the clinical predictor model to be on a computer screen during the sessions in the format we expect it to be used clinically. This proposed approach to using our model will be explored in the workshop and discussed in the context of the touch-point work, to determine what the potential issues, benefits, obstacles, and enablers are to the implementation, delivery, usefulness, and sustainability of this approach. We will ask the clinicians in the workshop to comment on the ease of use of the format and how they would like to access it (eg, an interactive formula on the BSGE website or embedded in guidelines). We will ask participants how the model should be used in secondary care consultations. We will also ask participants what is acceptable as a meaningful minimal change in pain score after surgery.

We will also ask the group to propose alternative approaches and solutions for any identified issues. For example, in our PPI work, it was suggested that the algorithm might be incorporated into a menstrual tracker app. As a follow-up from our study, we may develop a clinical trial of our clinical predictor model that builds on the workshop recommendations. This would need to consider the full patient pathway from primary care. Future trial considerations and the full patient pathway from primary to secondary care will therefore form a part of our workshop discussions.

### Ethics Approval and Consent to Participate

The main project involves the analysis of anonymized data sets, and thus does not require ethical review. We will need an ethical review for the workshop. As this will be held at the end of the study and no other processes are dependent on it, the ethical review application will be prepared after the project has commenced.

## Results

This project was funded in March 2018, approved by the Institutional Research Ethics Board in December 2019, and was in the phase of data analysis at the time the final revisions of this paper were made, with the results expected to be available in April 2021.

## Discussion

### Principal Strengths

This protocol defines the methods that will be applied to develop and externally validate our clinical prediction models to predict which women will benefit most from laparoscopic surgery resulting in reduction of pain or increased QoL. Previous models have focused on directly predicting endometriosis in patients with chronic pelvic pain and other symptoms, with limited success, or restricted their models to limited patient populations, with limited generalizability.

Our approach will address some of the challenges that other researchers have faced when attempting to improve care for women with chronic pelvic pain and endometriosis. As the prediction models focus on patient-perceived outcomes (QoL and pain), they will be more clinically relevant to the patient, and we are not limited by the wide range of definitions and treatments specific to endometriosis diagnosis, which often prevents external validation. The use of pre-existing data allows a comparatively quick and efficient development of the model with no unknown quantities, such as attrition and data completeness.

### Limitations

The use of pre-existing data collected as part of other projects means that we will be limited by the data as recorded and will have no input as to how and what information is collected. Nonetheless, we have been able to assess the extent of missing data and found it to be sufficiently low. Power considerations have been based on truly available data, and the large sample size, specifically in the BSGE data set, will allow us to investigate a wide range of prognostic factors.

A second limitation is the matching of variables in the development and external validation data sets. This challenge is common to many studies that use pre-existing external validation data sets. We have been comparing how data were collected in the data sets and found most variables to be compatible; however, not all factors that we may identify as prognostic will also be available in the validation data set, thereby limiting the list of prognostic factors that can be validated externally.

### Main Outputs and Access

This study will provide robustly developed and externally validated prediction models for postsurgery treatment success in women with suspected or confirmed endometriosis. The models will be generalized to a large range of women with this condition. To our knowledge, this project will be the first to predict who will benefit most from laparoscopic surgery resulting in reduction of pain or increased QoL and is therefore much needed. The prediction models will provide clinicians with a supporting tool for improving decision making in the diagnosis and treatment of women, thereby reducing unnecessary costs and harms associated with laparoscopic surgery.

Upon completion of the entire study, the models will be put in the public domain and will potentially be available for immediate use. The plan is that patient characteristics and clinical data can be entered into the formula by the user to calculate an individualized prediction of improvement after surgery. Presentation, implementation, and uptake within secondary care will be refined and finalized after the co-design workshops and beyond the lifespan of this project. For example, access could be in the form of a website or phone app. Our work with patient groups in the co-design workshops will give us invaluable direction on how to best advertise and deploy the prediction models, ensuring the greatest possible gain for patients and clinicians.

## Abbreviations

BSGE: British Society of Gynaecological Endoscopy
DE: deep endometriosis
LUNA: laparoscopic uterosacral nerve ablation
MEDAL: magnetic resonance imaging to establish diagnosis against laparoscopy
MRI: magnetic resonance imaging
NHS: National Health Service
PCTU: pragmatic clinical trials unit
PPI: patient and public involvement
PRISMA: Preferred Reporting Items for Systematic Reviews and Meta-Analyses
QoL: quality of life
RCT: randomized controlled trial
VAS: visual analog scale
